# Effects of Modified Atmosphere Packaging with Varied CO_2_ and O_2_ Concentrations on the Texture, Protein, and Odor Characteristics of Salmon during Cold Storage

**DOI:** 10.3390/foods11223560

**Published:** 2022-11-09

**Authors:** Yun-Fang Qian, Cheng-Cheng Liu, Jing-Jing Zhang, Per Ertbjerg, Sheng-Ping Yang

**Affiliations:** 1 College of Food Science & Technology, Shanghai Ocean University, Shanghai 201306, China; 2 Shanghai Engineering Research Center of Aquatic Product Processing & Preservation, Shanghai Ocean University, Shanghai 201306, China; 3 National Experimental Teaching Demonstration Center for Food Science and Engineering, Shanghai Ocean University, Shanghai 201306, China; 4 Department of Food and Nutrition, University of Helsinki, 00014 Helsinki, Finland

**Keywords:** salmon, modified atmosphere packaging, protein content, odor characteristics, correlation analysis

## Abstract

The effect of gas ratio on the growth of bacteria has been well demonstrated, but some adverse effects of modified atmosphere packaging (MAP) on seafoods have also been found. To provide a better understanding of the effects of CO_2_ and O_2_ concentrations (CO_2_ from 40% to 100% and O_2_ from 0% to 30%) in MAP on the texture and protein contents and odor characteristics of salmon during cold storage, the physiochemical, microbial, and odor indicators were compared with those without treatment (CK). Generally, MAP treatments hindered the increase of microbial counts, total volatile basic nitrogen, and TCA-soluble peptides, and decreased the water-holding capacity, hardness, springiness, and sarcoplasmic and myofibrillar protein contents. The results also indicated that 60%CO_2_/10%O_2_/30%N_2_ was optimal and decreased the total mesophilic bacterial counts by 2.8 log cfu/g in comparison with CK on day 12. In agreement, the concentration of CO_2_ of 60% showed the lowest myofibrillar protein degradation, and less subsequent loss of hardness. The electronic nose characteristics analysis indicated that 60%CO_2_/20%O_2_/20%N_2_ and 60%CO_2_/10%O_2_/30%N_2_ had the best effect to maintain the original odor profiles of salmon. The correlation analysis demonstrated that microbial growth had a strong relationship with myofibrillar and sarcoplasmic protein content. It can be concluded that 60%CO_2_/10%O_2_/30%N_2_ displayed the best effect to achieve the goal of preventing protein degradation and odor changes in salmon fillets.

## 1. Introduction

Salmon (*Salmo salar*) is an anadromous fish that is mainly produced in the Arctic and Atlantic Oceans and is favored by consumers worldwide. In recent years, salmon has become increasingly popular for its high nutritional value and good taste [1]. However, salmon is extremely vulnerable to spoilage during the period of storage and transportation. The spoilage is mainly dominated by microbial activities, consequently resulting in organoleptic rejection and economic loss [2].

To maintain the quality of salmon and prolong the shelf life, techniques are required with safety, eco-friendly, and convenient properties. Modified atmosphere packaging (MAP) is one of the most used physical techniques to prolong the shelf life of aquatic products by inhibiting microbial growth and delaying protein degradation [3,4]. The gas mixture in MAP is usually composed of carbon dioxide (CO_2_), nitrogen (N_2_), and oxygen (O_2_), in which CO_2_ is considered to have bacteriostatic and fungistatic properties, N_2_ to balance the gas composition and to prevent packaging collapse, and O_2_ to inhibit the growth of anaerobic microorganisms and reduce the formation of trimethylamine in fresh fish [5,6]. Most studies are focused on bacterial growth and microbial community [7,8,9], and it is considered that the bacteriostatic effect of MAP increases with an increasing concentration of CO_2_ [10]. Therefore, MAP with high concentrations of CO_2_ is generally applied for the chilled storage of animal products [11]. A modified atmosphere containing 96% CO_2_ was reported to limit microbial growth in Atlantic salmon [7]. In another study, freezing treatment with an atmosphere of 60% CO_2_ and 40% N_2_ could not only inhibit the growth of *Photobacterium phosphoreum*, but could also inhibit the production of biogenic amines [2]. Recent studies also revealed that high concentrations of CO_2_ displayed some adverse effects of MAP on the sensory and physical characteristics of seafoods [12]. However, only few studies focused on the mechanisms of how the atmosphere composition affects the texture, protein, and odor characteristics of salmon fillets. Due to the importance of sensory quality affecting consumers’ decisions, it is necessary to systematically study the effect of different concentrations of CO_2_ and O_2_ on the muscle and odor quality of salmon.

Therefore, a better understanding of how the atmosphere affects the quality deterioration of salmon and how to optimize the gas mixture of MAP is needed. The aim of this study was to investigate the influences of different concentrations of CO_2_ and O_2_ on the texture, muscle protein, and odor characteristics of salmon at 4 °C, and the correlation between texture, protein content, and microbial growth was also analyzed.

## 2. Materials and Methods

### 2.1. Sample Preparation

**Figure 1:**
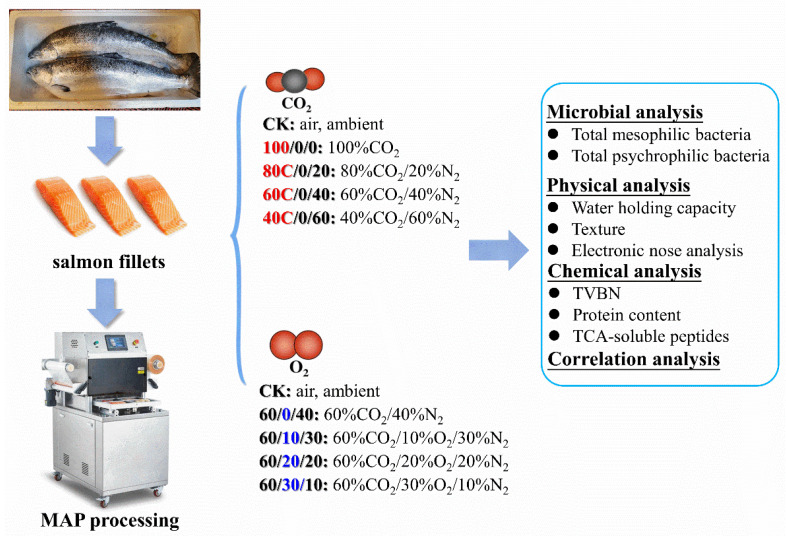
A schematic overview of the experimental study.

The original salmon (*Salmo salar*) samples (each one about 6 kg) were purchased from a local supermarket (place of origin: Norway), where the salmon samples were beheaded, skinned, and deboned by skillful staff in the workshops of the supermarket. The fish was cut into pieces each weighing about 100 g, and 49 pieces were selected randomly for further study. Then they were transferred to the laboratory in an incubator with ice slurry within one hour. After arrival, the samples were washed in cold water, and then the drips were drained off before putting them into polyamide/polyethylene pouches (PA/PE, Xilong Packaging Co., Ltd., Shijiazhuang, China; size: 32×22 cm; thickness: 0.32 mm; oxygen permeability (O_2_P): 7 cm^3^/(m^2^⋅24 h⋅0.1 MPa)) randomly. To investigate the effect of CO_2_ and O_2_ on the texture, protein, and odor characteristics of salmon, the pouches were separated into eight different batches, settled as following (Figure 1): (1) CK: air, ambient; (2) 100/0/0: 100%CO_2_; (3) 80/0/20: 80%CO_2_/20%N_2_; (4) 60/0/40: 60%CO_2_/40%N_2_; (5) 40/0/60: 40%CO_2_/60%N_2_; (6) 60/10/30: 60%CO_2_/10%O_2_/30%N_2_; (7) 60/20/20: 60%CO_2_/20%O_2_/20%N_2_; (8) 60/30/10: 60%CO_2_/30%O_2_/10%N_2_.

The MAP pouches were emptied by vacuum, and then the gas mixtures were flushed into them, and, finally, they were heat-sealed by gas-flushing equipment (Model BQ-360W, Shanghai Qingba Food Packaging Machinery Co., Ltd., Shanghai, China) with a gas volume-to-product (G/P) ratio of 3:1. Finally, all samples were stored at 4 °C for 12 days.

### 2.2. Microbiological Analysis

The bacterial counts were measured according to the method described by Yu et al. [13] with some modifications. A sample (25 g) was mixed with 225 mL sterilized salines in a sterile plastic bag and homogenized for 1 min to 2 min. After dilution in 10-folded series, 1 mL of an appropriate dilution was added into Plate Count Agar (PCA, No. HB0101, Qingdao Hope Biol-Technology Co., Ltd., Qingdao, China) with 5% NaCl (Sangon Biotech Co., Ltd. Shanghai, China). The total mesophilic bacterial count (TMB) was incubated on PCA at 30 °C for two days. Next, the total psychrophilic bacteria (TPB) were cultivated at 4 °C for ten days.

### 2.3. Determination of Total Volatile Basic Nitrogen (TVB-N)

The total volatile basic nitrogen (TVB-N) value was measured according to the previous studies by an Automatic Kjeldahl Apparatus (KjeltecTM8400; FOSS Quality Assurance Co., Ltd., Copenhagen, Denmark) [14]. The value of the TVB-N was expressed as mg N/100 g salmon. Each sample was tested in triplicate.

### 2.4. Determination of Water-Holding Capacity (WHC)

The measurement method in this study was developed by Bao et al. [15] and Liu et al. [16]. In brief, the sample was cut into small pieces (weight about 2 g, recorded as M_1_), and then the sample was weighed again after centrifugation at 1590 g for 10 min, and the value was recorded as M_2_. Triplicate samples were calculated by the following formula:

(1) $$\mathrm{WHC}=\frac{M_{2}}{M_{1}}\times 100\%$$

### 2.5. Texture Profile Analysis (TPA)

The texture test was conducted according to Wang et al. [17] by a TA.XT Plus analyzer (Stable Micro Systems, Ltd., Surrey, UK) equipped with a P/5 probe. The fresh sample was cut into small squares (20 mm × 20 mm × 10 mm). The parameters of hardness, chewiness, springiness, and gumminess were acquired at a speed of 1 mm/s and a sample deformation of 50%. Each sample was tested six times.

### 2.6. Determination of TCA-Soluble Peptide Content

The TCA-soluble peptide content determination was performed according to the method of Zhuang et al. [18] with some modifications. The minced sample (2 g) was homogenized with 18 mL 5% (*w/v*) trichloroacetic acid (TCA) solution, and incubated at 4 °C for 1 h. Then the sample was centrifuged at 10,000× *g* for 5 min. The supernatant was collected. The content of TCA-soluble peptide was determined by Lowry’s method, and was expressed as μmol tyrosine/g muscle. Each sample was tested three times.

### 2.7. Determination of Protein Content

The myofibrillar protein was extracted according to the method of Lv et al. [19]. About 2 g minced fish sample was homogenized with 20 mL Tris-maleate buffer A (20 mmol/L pH 7.0, 0.05 mol/L KCl) at 4 °C. Then, the solution was centrifuged for 15 min at a speed of 10,000× *g.* The supernatant containing the sarcoplasmic protein was collected. The precipitate was homogenized with 20 mL Tris-maleate buffer B (20 mmol/L, pH 7.0, containing 0.6 mol/L KCl). The solution was incubated for 1 h at 4 °C. After centrifugation at 10,000× *g* for 15 min, the supernatant containing the salt-soluble myofibrillar protein was obtained. The Biuret method was used to determine the content of protein using bovine serum albumin as standard, and the result was expressed as mg/g. All samples were repeated three times.

### 2.8. Electronic Nose Analysis

The electronic nose (Alpha MOS, Toulouse, France) consisted of an auto-sampling device, a detector unit containing 18 metal oxide sensors (MOSs), and pattern recognition software for data recording, and interpretation was used to provide flavor attributes of the salmon during storage. The electronic nose sensor signals were measured by the method of Zhang et al. [20]. Each sample was mixed with 0.18 g/mL NaCl solution at a mass ratio of 1:1. Before the test, the salmon sample was put into a 20 mL airtight vial and incubated at 45 °C for 50 min. The test parameters were settled with the measurement time for 120 s and the standby procedure for 500 s. All samples were repeated six times, and only the last five pieces of data of each sample were used for further analysis to eliminate the impact from the previous group with different treatments. The signals were further analyzed by the system (Alpha MOS, Toulouse, France) to distinguish discrepancies between the odor profiles of the groups with different gas ratios on different days, and the principal component analysis diagrams (PCA) were outputted by the system.

### 2.9. Statistic Analysis

The analyses described above were carried out on days 0, 2, 4, 6, 8, 10, and 12. The one-way ANOVA procedure with the package type ((1) CK: air, ambient; (2) 100/0/0: 100%CO_2_; (3) 80/0/20: 80%CO_2_/20%N_2_; (4) 60/0/40: 60%CO_2_/40%N_2_; (5) 40/0/60: 40%CO_2_/60%N_2_; (6) 60/10/30: 60%CO_2_/10%O_2_/30%N_2_; (7) 60/20/20: 60%CO_2_/20%O_2_/20%N_2_; (8) 60/30/10: 60%CO_2_/30%O_2_/10%N_2_.), followed by Duncan’s test, was carried out for multiple comparisons by the SPSS 20.0 software (SPSS Version 20.0, Inc., Chicago, IL, USA), and significance was judged at 0.05. The diagrams of the changes in the indicators of samples under different atmospheres against storage time were designed by the Origin2018 software (OriginLab, Northampton, MA, USA). The results are expressed as means ± standard deviation. The relationship between indicators was assessed by Pearson correlation analysis, and the diagram was also designed by the Origin2018 software (OriginLab, Northampton, MA, USA).

## 3. Results and Discussion

### 3.1. Microbial Growth

**Figure 2:**
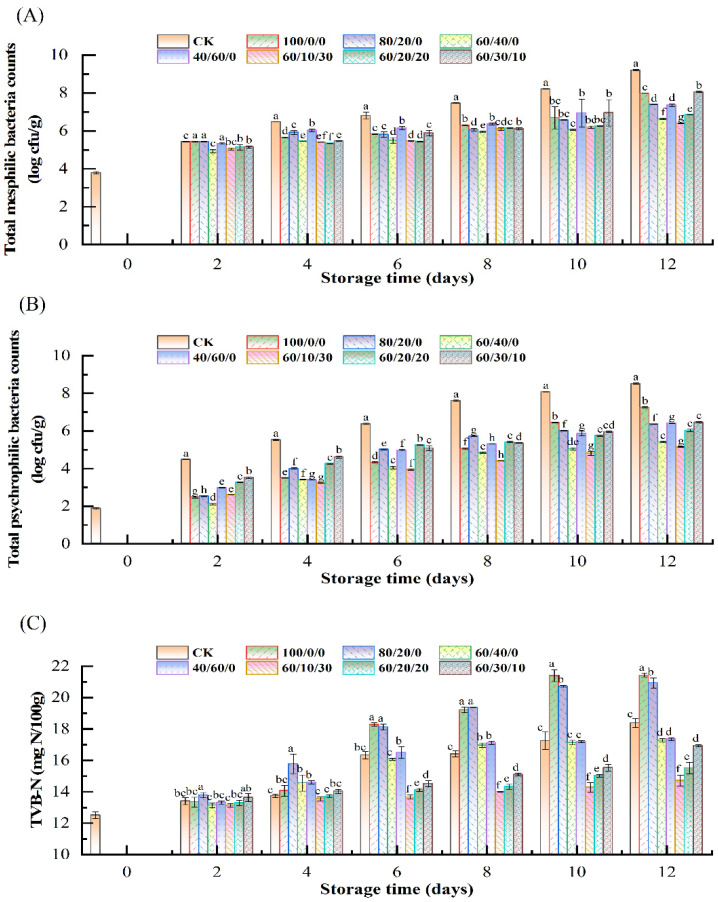
Changes of total mesophilic bacteria (**A**), the total psychrophilic bacteria (**B**), and TVB-N (**C**) in salmon fillets under MAP containing different CO_2_/O_2_/N_2_ ratios during storage at 4 °C. Different lowercase letters (a–h) represent significant differences (*p* < 0.05) between different groups at the same storage period.

The effect of different concentrations of CO_2_ and O_2_ on the growth of mesophilic bacteria in salmon is shown in Figure 2A. The initial TMB count was 3.79 log cfu/g, indicating that the sample was fresh. The TMB counts increased continuously during storage in all packaging conditions. All MAP groups had significantly lower TMB counts than the sample under air after 4 days of storage. The TMB counts of the control were close to 7 log cfu/g on day 6 and reached 9.20 log cfu/g at the end of storage, whereas most samples with 60% CO_2_ did not exceed the threshold during the whole period, except for 60%CO_2_/30%O_2_/10%N_2_. It is found in Figure 2A that MAP with CO_2_ higher than 40% effectively inhibited the growth of the mesophilic bacteria, which was also stated by Fernandez et al. [10]. Similarly, it is reported that MAP with CO_2_ higher than 40% could prolong the shelf life of Pacific white shrimp effectively [21]. However, higher CO_2_ did not always lead to lower bacterial counts, because 100%CO_2_ and 80%CO_2_/20%N_2_ had higher TMB counts than 60%CO_2_/40%N_2_. It was hypothesized that high concentrations of CO_2_ might help to increase the solubility of the muscle protein, consequently providing nutrients for bacterial growth [22]. As shown in Figure 2A, different concentrations of O_2_ also affected the increase of TMB counts. It is known that the existence of O_2_ can slow the growth of anaerobic bacteria [23]. However, our study showed that the TMB counts of 60%CO_2_/30%O_2_/10% N_2_ were higher than 60%CO_2_/40%N_2_, 60%CO_2_/10%O_2_/30%N_2_, and 60%CO_2_/20%O_2_/20%N_2_, indicating that 30%O_2_ could promote bacterial growth. Therefore, a MAP with O_2_ higher than 20% was not recommended according to the results.

The changes of total psychrophilic bacteria count of salmon packaged under different concentrations of CO_2_ and O_2_ are shown in Figure 2B. The TPB counts of the control rose from 1.87 log cfu/g to 8.52 log cfu/g during storage. Compared with the results of TMB, it is demonstrated that the proportion of psychrophilic bacteria to mesophilic bacteria was low initially, but increased during storage, in agreement with the study of Yang et al. [24]. The pattern change of TPB was similar to TMB, and 60%CO_2_/10%O_2_/30%N_2_ had the lowest TPB counts. Therefore, 60%CO_2_/10%O_2_/30%N_2_ was recommended according to the results of bacterial growth.

### 3.2. Changes of TVB-N Content

Animal products rich in protein, amino acid, and other nutrients can easily be decomposed into ammonia and other basic volatile substances, which could be detected as total volatile basic nitrogen (TVB-N) content [25]. Figure 2C display the changes of TVB-N content in salmon under different modified atmosphere packaging. The TVB-N of all groups increased gradually during storage. The samples of 100%CO_2_ and 80%CO_2_/20%N_2_ had higher TVB-N contents than other groups after 6 days of storage. The results indicated that CO_2_, when higher than 80%, might promote the accumulation of TVB-N. Compared with the changes of bacterial counts, it is found that the changes of TVB-N were consistent with the bacterial counts. It is also observed that 60%CO_2_/10%O_2_/30%N_2_, 60%CO_2_/20%O_2_/20%N_2_, and 60%CO_2_/30%O_2_/10%N_2_ had lower TVB-N contents than CK and 60%CO_2_/40%N_2_, indicating that O_2_ was also beneficial for inhibiting the generation of TVB-N. However, the inhibitory effect of O_2_ decreased with its increasing concentration. This phenomenon might be contributed to the microbial growth [26].

### 3.3. Changes of Water-Holding Capacity

**Figure 3:**
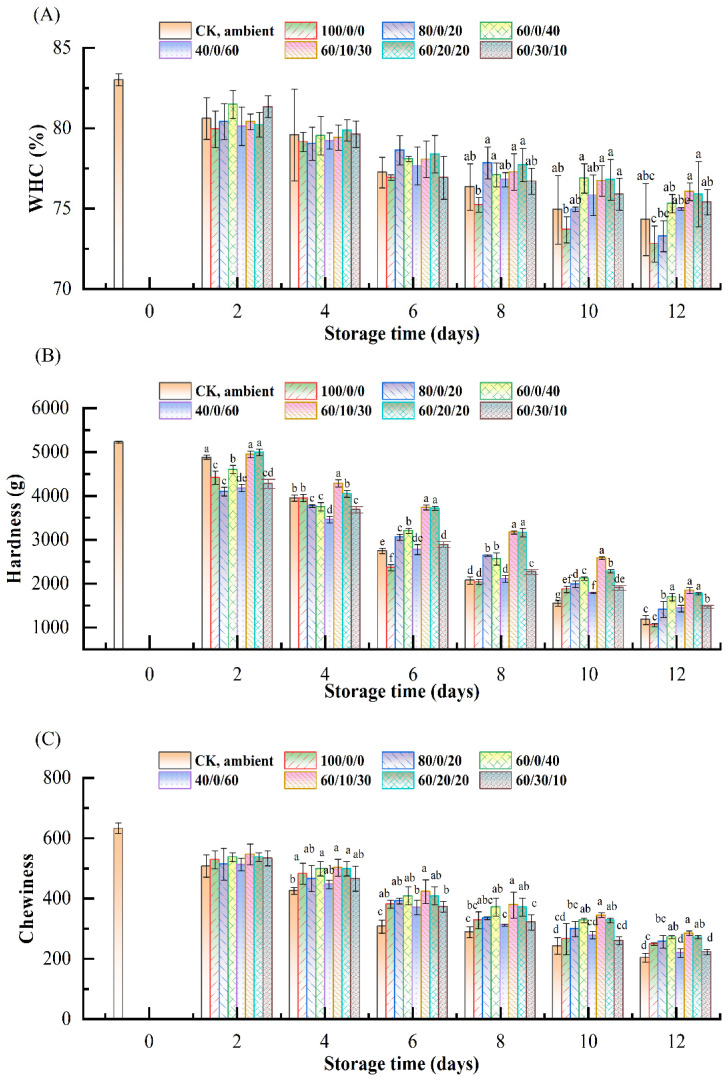

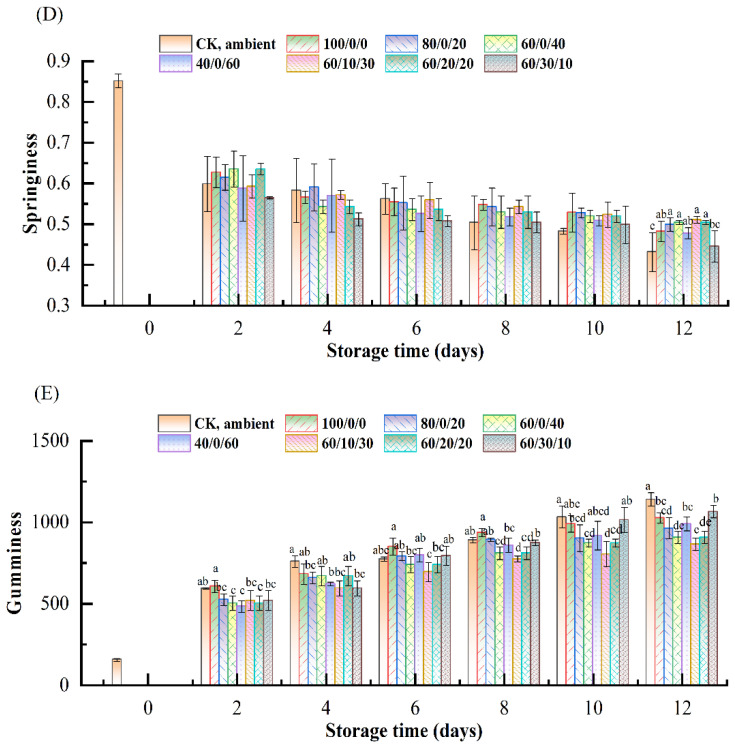
Changes of WHC (**A**), hardness (**B**), chewiness (**C**), springiness (**D**), and gumminess (**E**) of salmon fillets under MAP containing different CO_2_/O_2_/N_2_ ratios at 4 °C. Different lowercase letters (a–d) represent significant differences (*p* < 0.05) between different groups at the same storage period.

The water-holding capacity (WHC) of all samples displayed a decreasing tendency as the storage time increased (Figure 3A). The WHC of CK decreased from 83.01% to 74.31%. It was found that CK had lower WHC than MAP groups except for 100%CO_2_ and 80%CO_2_/20%N_2_. An adverse effect of CO_2_ on WHC was observed in this study, which might be associated with the solubilization of CO_2_ in the muscle, leading to muscle shrinkage and a decreased ability to retain water [26]. Another reason might be that CO_2_ promoted proteolytic degradation. The samples of 60%CO_2_/10%O_2_/30%N_2_ and 60%CO_2_/20%O_2_/20%N_2_ had the highest level of WHC, indicating low concentrations of O_2_ would be beneficial for maintaining the WHC of salmon fillets. However, atmospheres with O_2_ concentrations higher than 20% might accelerate protein oxidation and result in decreased WHC [27].

### 3.4. Changes of Texture Properties

The texture profiles of salmon during MAP storage are shown in Figure 3B–E. As shown in Figure 3B, the hardness of salmon reduced gradually during storage time, which may be attributed to the activity of endogenous and microbial proteolytic enzymes [28]. The hardness of CK samples was at the lowest level at the later storage times. As shown in Figure 3B, it was found that the hardness of sample 100/0/0 was lower than other MAP groups, especially on day 6, indicating that 100% CO_2_ had an adverse effect on the hardness of salmon. However, CO_2_ at 80% and 60% inhibited the decrease of hardness. The modified atmosphere with different concentrations of O_2_ also affected the hardness (Figure 3B). The samples of 60%CO_2_/10%O_2_/30%N_2_ and 60%CO_2_/20%O_2_/20%N_2_ had higher hardness than other groups, and the hardness of 60%CO_2_/30%O_2_/10%N_2_ was relatively lower, which was coherent with the results of microbial growth.

The results of the chewiness and springiness of salmon during storage are displayed in Figure 3C,D, respectively. It was observed that both the chewiness and springiness decreased during storage. The samples of 60%CO_2_/10%O_2_/30%N_2_ and 60%CO_2_/20%O_2_/20%N_2_ had the highest values of chewiness, meaning a lower level of O_2_ would be suitable for maintaining the chewiness of salmon. The initial springiness of fresh salmon was 0.85, and the control decreased to 0.56 on day 6, and even reached 0.43 on day 12. A sharp decrease of springiness could be observed on day 2, and the samples of 60%CO_2_/10%O_2_/30%N_2_ and 60%CO_2_/10%O_2_/30%N_2_ held the highest springiness on day 12. The results indicated that MAP with 60% CO_2_ and O_2_ below 20% maintained the chewiness and springiness of salmon fillets most effectively, and higher CO_2_ and O_2_ provided an adverse effect.

The gumminess of salmon fillets increased continuously with the extension of storage time (Figure 3E), which likely was mainly due to the microbial growth [29]. Significant differences were observed between CK and MAP groups at the end of storage, probably because MAP effectively inhibited microbial growth and reproduction. Both CO_2_ and O_2_ had some impact on the gumminess of salmon fillets. Compared with the groups with different gas mixtures, the results indicated that 60% CO_2_ was more appropriate to slow the increase of gumminess, whereas 10% O_2_ had a better effect than 0%, 20%, and 30%. Therefore, the concentrations of CO_2_ should not be higher than 60%, and a low level of O_2_ (~10%) was more beneficial for maintaining the gumminess of salmon fillets.

### 3.5. Changes of TCA-Soluble Peptide Content

**Figure 4:**
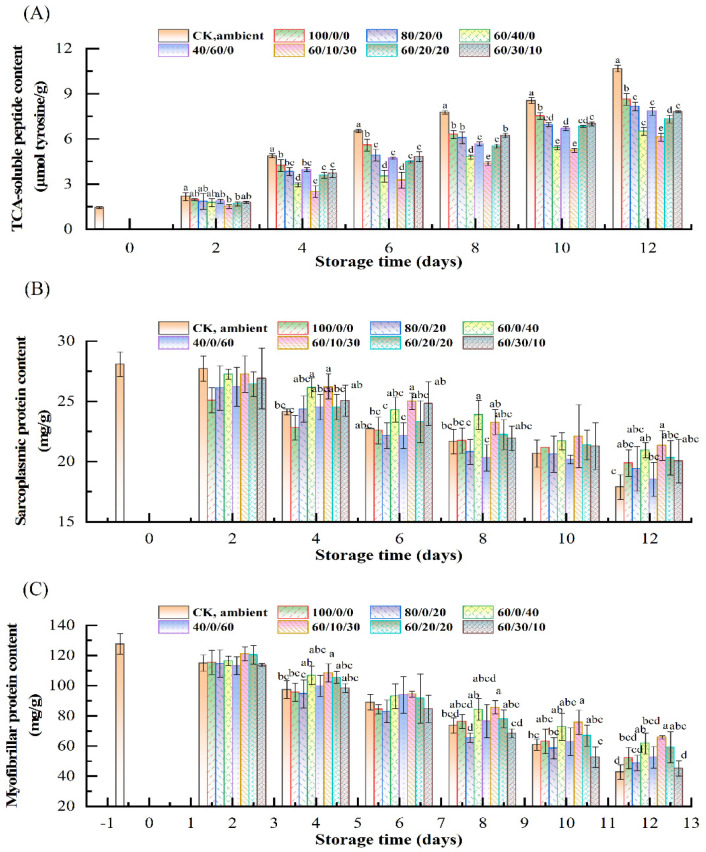
Changes of TCA-soluble peptides (**A**), sarcoplasmic protein content (**B**), and myofibrillar protein content (**C**) of salmon under MAP containing different CO_2_/O_2_/N_2_ ratios at 4 °C. Different lowercase letters (a–c) represent significant differences (*p* < 0.05) between different groups at the same storage period.

The content of TCA-soluble peptides is an important indicator of protein degradation in fish flesh. As shown in Figure 4A, the TCA-soluble peptide content of the fresh sample was 1.44 μmol tyrosine/g. With increased storage time, the content of TCA-soluble peptides of CK increased to 10.66 μmol tyrosine/g on day 12. The content of TCA-soluble peptides in 100C was higher than other MAP groups at the end of storage. This phenomenon is probably related to the disruption of muscle cells by CO_2_, leading to an increase of proteolytic activity. The content of TCA-soluble peptides of MAP groups with different concentrations of O_2_ increased much more slowly. Among them, the sample of 60%CO_2_/10%O_2_/30%N_2_ had the lowest TCA-soluble peptide content, and, thus, only reached 6.12 μmol tyrosine/g on day 12. It is reported that the protein degradation is not only induced by endogenous enzymes [30,31], but is also caused by spoilage bacteria [32]. Therefore, the lower content of TCA-soluble peptides found in the sample of 60%CO_2_/10%O_2_/30%N_2_ could be mainly attributed to its antimicrobial effect, consistent with the results of microbial growth.

### 3.6. Changes of Protein Contents

Salmon flesh is mainly composed of sarcoplasmic and myofibrillar protein, which account for almost 85% of the total protein contents [33,34]. Therefore, the changes of sarcoplasmic and myofibrillar protein contents were measured and the results are displayed in Figure 4B,C.

The results show that the sarcoplasmic protein (SP) contents in all packaging systems decreased during storage. It is well known that sarcoplasmic proteins, to a large extent, are composed of small and intermediate size proteins, such as some water-soluble enzymes [35]. Our results showed that samples stored without oxygen under 100%, 80%, and 40% CO_2_ had lower contents of sarcoplasmic protein than those under 60% CO_2_, indicating that 60% CO_2_ balanced the anti-bacterial activity and anti-hydrolysis activity. Moreover, the contents of sarcoplasmic protein also decreased with the increasing O_2_ concentrations, and 60%CO_2_/10%O_2_/30%N_2_ and 60%CO_2_/40%N_2_ had the highest contents of sarcoplasmic protein compared to other groups. This phenomenon should be attributed to the lower microbial metabolic activity in these samples.

As shown in Figure 4C, the content of myofibrillar protein (MP) soluble at high ionic strength in all groups decreased gradually, especially in CK. Myofibrillar protein is known as an important component of the muscle fiber, which is composed of salt-soluble proteins such as myosin, actin, and troponins [36]. The initial content of myofibrillar protein in fresh salmon was 128 mg/g, but it decreased sharply to 89 mg/g after 6 days of storage, and even to 43 mg/g at the end of storage in CK. The decrease of myofibrillar protein content should be attributed to the aggregation, degradation, and oxidation processes [37]. The content of myofibrillar protein in samples stored under 60% CO_2_ was maintained at a higher level. This was probably mainly because this MAP system could effectively maintain the protein content by inhibiting microbial growth [38].

### 3.7. Changes of Odor Characteristics

**Figure 5:**
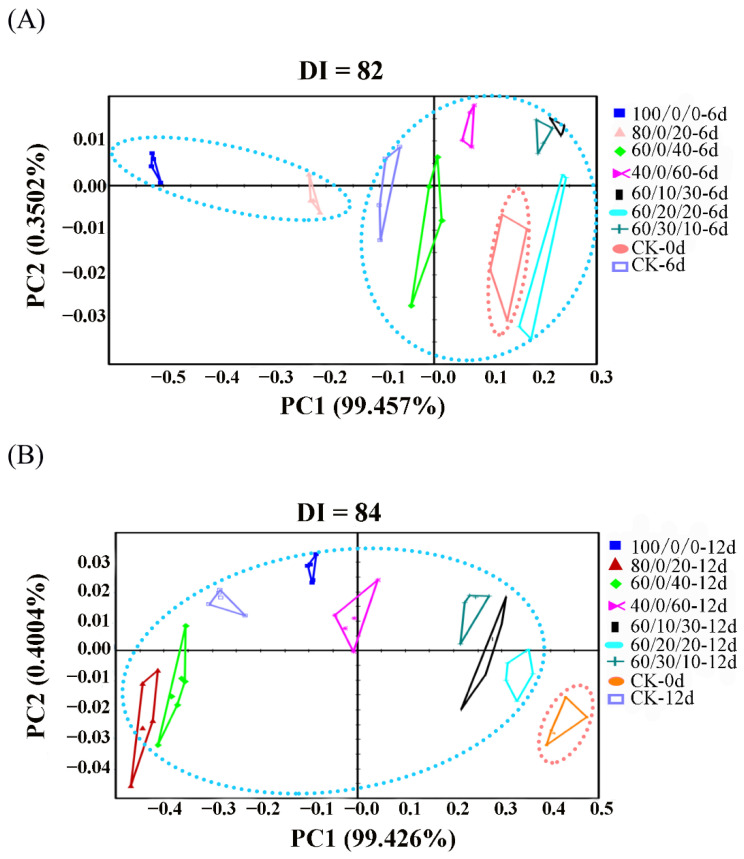
PCA plots of the volatile odor characteristics by electronic analysis of salmon under MAP containing different CO_2_/O_2_/N_2_ ratios at 4 °C on day 6 (**A**) and day 12 (**B**) in comparison with CK-0d.

In this study, the electronic nose was used to analyze the odor characteristics of salmon fillets during the MAP storage period. The principal component analysis (PCA) was used to distinguish electronic nose sensor response signals of the samples on day 6 and day 12 (Figure 5). The results are presented in the form of a two-dimensional scatter diagram on the basis of the original information system and feature vectors in linear classification after dimensionality reduction. The different index (DI) was used to reflect the overall differentiation effect of odor characteristics, and its range was generally from 0 to 100, with 80 as the distinguishing threshold [39,40]. The figures also indicate that the first two principal component axes (PC1 and PC2) and the spatial distribution areas reflected the differences between samples. The DI of Figure 5A,B were 82 and 84, respectively. PC1 and PC2 for Figure 5A were 99.46% and 0.35%, whereas those for Figure 5B were 99.43% and 0.40%, respectively. Therefore, the PCA results covered almost all of the varied information, and the results could be distinguished effectively. As shown in Figure 5A, the plots of salmon fillets stored under 100%CO_2_ after 6 days of storage were distant from the initial samples, the control, and other MAP groups, whereas the sample stored under 60%CO_2_/20%O_2_/20%N_2_ was clustered close to CK-0d (fresh sample), suggesting similar odor profiles. Therefore, 100%CO_2_ atmosphere packaging might change the intrinsic odor characteristics of salmon. After 12 days of storage, the plots of all samples were more distant from the fresh sample (CK-0d) (Figure 5B), and the plots of 60/20/20-12d and 60/10/30-12d were much closer to CK-0d than other samples. Therefore, 60%CO_2_/20%O_2_/20%N_2_ and 60%CO_2_/10%O_2_/30%N_2_ displayed a better effect to maintain the original odor profiles of salmon. The changes of odor in samples stored under higher concentrations of CO_2_ and lower concentrations of O_2_ might be attributed to the growth of lactic bacteria, which are anaerobic, gram-positive, and CO_2_-resistant species, and can provide sour off-odors [41].

### 3.8. Correlation Analysis

**Figure 6:**
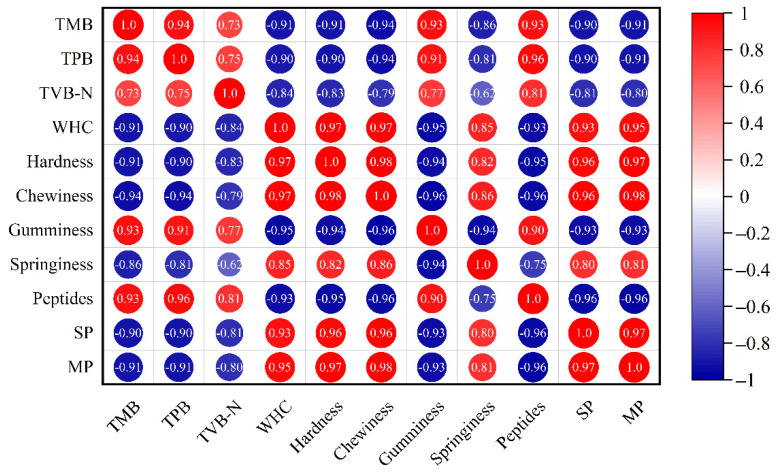
Heatmap of Pearson correlation between the TMB, TPB, TVB-N, WHC, hardness, chewiness, gumminess, springiness, TCA-soluble peptides, sarcoplasmic and myofibrillar protein content in salmon under modified atmosphere packaging. The circle size represents the absolute value of the correlation coefficient, where red represents positive correlation, and blue represents negative correlation.

The results of the correlation analysis between protein changes, microbial growth, and texture properties are shown in Figure 6. The results show that TMB, TPB, the contents of TCA-soluble peptides, myofibrillar protein, and sarcoplasmic protein all had significant correlations with hardness, springiness, chewiness, gumminess, and WHC. The absolute value of Pearson-related coefficients between TMB/TPB and myofibrillar/sarcoplasmic protein/TCA-soluble peptides was even higher than 0.8, indicating that the microbial growth had a strong relationship with the myofibrillar and sarcoplasmic protein content. Moreover, WHC seemed to have a stronger relationship with chewiness, hardness, and myofibrillar protein content than gumminess, springiness, sarcoplasmic protein, and TCA-soluble peptide content. Therefore, the intact structure of the myofibrillar protein was essential for maintaining WHC, chewiness, and hardness.

**Figure 7:**
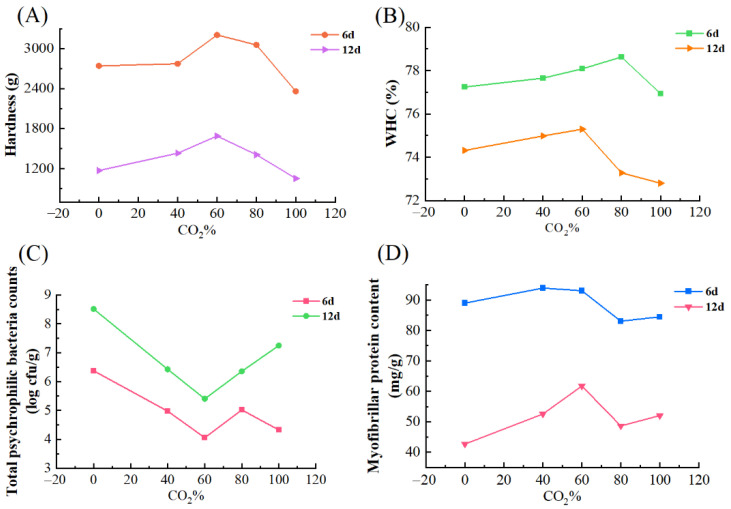
Relationship between CO_2_ concentrations and hardness (**A**), WHC (**B**), TPB (**C**), and myofibrillar protein content (**D**) of salmon fillets stored under modified atmosphere packaging containing O_2_ of 0% at 4 °C on 6 d and 12 d.

The effect of CO_2_ concentrations on the hardness, WHC, myofibrillar protein content, and TPB of salmon fillets are shown in Figure 7. The values plotted were on 6 d and 12 d with CO_2_ concentrations from 40% to 100% and 0% O_2_. It could be observed that hardness increased with an increasing CO_2_ concentration when the CO_2_ concentration was up to 60%. However, when the CO_2_ concentration was higher than 60%, increasing CO_2_ would result in decreased hardness. A similar tendency was found in WHC and myofibrillar protein content. It is hypothesized that high CO_2_ contributed to the changes in the microstructure of the muscle [42]. The increase of CO_2_ concentration led to a lower count of TPB when CO_2_ was below 60%, which could be attributed to the antibacterial effect of CO_2_. However, CO_2_ higher than 60% had an adverse effect on TPB. Therefore, 60% CO_2_ was recommended to maintain the quality of salmon fillets.

## 4. Conclusions

In this study, the results showed that increased CO_2_ from 40% to 100% and decreased O_2_ from 30% to 0% could effectively inhibit the growth of mesophilic and psychrophilic bacteria of salmon fillets from day 4 to day 10. However, 100% CO_2_ and 80% CO_2_ also led to a reduction of hardness, WHC, and myofibrillar protein content in comparison with 60% CO_2_. Moreover, CO_2_ higher than 80% might also promote the accumulation of TVB-N in salmon. Additionally, O_2_ higher than 20% also possibly affected protein degradation and subsequent muscle texture deterioration. The E-nose analysis indicated that 60%CO_2_/20%O_2_/20%N_2_ and 60%CO_2_/10%O_2_/30%N_2_ displayed the best effect to maintain the original odor profiles of salmon. The microbial growth had a strong negative correlation with myofibrillar and sarcoplasmic protein content. A stronger positive relationship between WHC and chewiness, hardness, and myofibrillar protein content was also found in the results. In conclusion, the content of myofibrillar protein is essential for the texture and water-holding capacity. Accordingly, a high CO_2_ content of 60% and low O_2_ of 10% in MAP is recommended to maintain the quality of salmon.

## Data Availability

Data are contained within the article.
